# Supplementary material for: Targeted pathogen profiling of ancient feces reveals common enteric infections in the Rio Zape Valley, 725–920 CE
Source: PLoS One. 2025 Oct 22;20(10):e0318140. doi: 10.1371/journal.pone.0318140 (PMC12543138; doi:10.1371/journal.pone.0318140)
Supplement: S1 Table — (DOCX) [file pone.0318140.s001.docx]

**Table S1.** Primer and probe sequences for qPCR assays on the custom TAC

NOTE: Though the TAC contained RNA targets, only the DNA targets were used in this study

| **Pathogen** | **Gene** | **Primer or probe sequence (5' - 3')** | **Ref** | **Amplicon**  **Length (bp)** |
| --- | --- | --- | --- | --- |
| *Campylobacter jejuni*/*C. coli* | cadF | Fwd: CTGCTAAACCATAGAAATAAAATTTCTCAC |  | 222 |
|  |  | Rev: CTTTGAAGGTAATTTAGATATGGATAATCG | [1] |  |
|  |  | Probe: CATTTTGACGATTTTTGGCTTGA |  |  |
| *C. difficile* | tcdB | Fwd: GGTATTACCTAATGCTCCAAATAG |  |  |
|  |  | Rev: TTTGTGCCATCATTTTCTAAGC | [1] | 88 |
|  |  | Probe: CCTGGTGTCCATCCTGTTTC |  |  |
| EAEC (aaiC) | *aaiC* | Fwd: ATTGTCCTCAGGCATTTCAC |  |  |
|  |  | Rev: ACGACACCCCTGATAAACAA | [1] | 216 |
|  |  | Probe: TAGTGCATACTCATCATTTAAG |  |  |
| EAEC (aatA) | *aatA* | Fwd: CTGGCGAAAGACTGTATCAT |  |  |
|  |  | Rev: TTTTGCTTCATAAGCCGATAGA | [1] | 238 |
|  |  | Probe: TGGTTCTCATCTATTACAGACAGC |  |  |
| STEC (stx1) | *stx1* | Fwd: ACTTCTCGACTGCAAAGACGTATG |  |  |
|  |  | Rev: ACAAATTATCCCCTGWGCCACTATC | [1] | 133 |
|  |  | Probe: 56FAM/CTCTGCAATAGGTACTCCA/3MGB-NFQ/ |  |  |
| STEC (stx2) | *stx2* | F, CCACATCGGTGTCTGTTATTAACC |  |  |
|  |  | R, GGTCAAAACGCGCCTGATAG | [1] | 94 |
|  |  | P, 5VIC/TTGCTGTGGATATACGAGG/3MGB-NFQ/ |  |  |
| EPEC (eae) | *eae* | F, CATTGATCAGGATTTTTCTGGTGATA |  |  |
|  |  | R, CTCATGCGGAAATAGCCGTTA | [1] |  |
|  |  | P, 56FAM/ATACTGGCGAGACTATTTCAA/3MGB-NFQ/ |  | 103 |
| EPEC (bfpA) | *bfpA* | F, TGGTGCTTGCGCTTGCT |  |  |
|  |  | R, CGTTGCGCTCATTACTTCTG | [1] | 111 |
|  |  | P, 5VIC/CAGTCTGCGTCTGATTCCAA/3MGB-NFQ/ |  |  |
| ETEC LT | *LT* | F, TTCCCACCGGATCACCAA |  |  |
|  |  | R, CAACCTTGTGGTGCATGATGA | [1] | 63 |
|  |  | P, CTTGGAGAGAAGAACCCT |  |  |
| ETEC ST | *ST* | Fh, GCTAAACCAGYAGRGTCTTCAAAA |  | 148 |
|  |  | Fp, TGAATCACTTGACTCTTCAAAA |  | 137 |
|  |  | Rh, CCCGGTACARGCAGGATTACAACA | [1] |  |
|  |  | Rp, GGCAGGATTACAACAAAGTT |  |  |
|  |  | Ph, 6VIC/TGGTCCTGAAAGCATGAA/3MGB-NFQ/ |  |  |
|  |  | Pp, 6VIC/TGAACAACACATTTTACTGCT/3MGB-NFQ/ |  |  |
| EIEC/*Shigella* | *ipaH* | F, CCTTTTCCGCGTTCCTTGA |  | 65 |
|  |  | R, CGGAATCCGGAGGTATTGC | [1] |  |
|  |  | P, 56FAM/CGCCTTTCCGATACCGTCTCTGCA/3MGB-NFQ/ |  |  |
| *Salmonella* | *ttr* | Fwd: CTCACCAGGAGATTACAACATGG |  | 78 |
|  |  | Rev: AGCTCAGACCAAAAGTGACCATC | [1] |  |
|  | MGB probe | Probe: CACCGACGGCGAGACCGACTTT |  |  |
| *E. coli* O157 | *rfbE* | Fwd: TTTCACACTTATTGGATGGTCTCAA |  |  |
|  |  | Rev: CGATGAGTTTATCTGCAAGGTGAT | [1] | 89 |
|  |  | Probe: CTCTCTTTCCTCTGCGGTCCT |  |  |
| *Cryptosporidium* | *18S* | Fwd: GGGTTGTATTTATTAGATAAAGAACCA |  |  |
|  |  | Rev: AGGCCAATACCCTACCGTCT | [1] | 126 |
|  |  | Probe: TGACATATCATTCAAGTTTCTGAC |  |  |
| *Giardia* spp. | *18S* | Fwd: GACGGCTCAGGACAACGGTT |  |  |
|  |  | Rev: TTGCCAGCGGTGTCCG | [1] | 63 |
|  |  | Probe: CCCGCGGCGGTCCCTGCTAG |  |  |
| *E. histolytica* | *18S* | Fwd: ATTGTCGTGGCATCCTAACTCA |  |  |
|  |  | Rev: GCGGACGGCTCATTATAACA | [1] | 175 |
|  |  | Probe: TCATTGAATGAATTGGCCATTT |  |  |
| *Entamoeba* spp. | *18S rRNA* | Fwd: AAACGATGTCAACCAAGGATTG |  |  |
|  |  | Rev: TCCCCCTGAAGTCCATAAACTC | [1] | 135 |
|  |  | Probe: CCTTGTTCAGAACTTAAAGAGAAA |  |  |
| *Ascaris* | *ITS1* | Fwd: GCCACATAGTAAATTGCACACAAAT |  |  |
|  |  | Rev: GCCTTTCTAACAAGCCCAACAT | [1] | 134 |
|  |  | Probe: TTGGCGGACAATTGCATGCGAT |  |  |
| *Trichuris* | *18S rRNA* | Fwd: TTGAAACGACTTGCTCATCAACTT |  | 77 |
|  |  | Rev: CTGATTCTCCGTTAACCGTTGTC | [1] |  |
|  |  | Probe: CGATGGTACGCTACGTGCTTACCATGG |  |  |
| *Necator americanus* | ITS-2 | Fwd: CTGTTTGTCGAACGGTACTTGC |  | 102 |
|  |  | Rev: ATAACAGCGTGCACATGTTGC | [1] |  |
|  |  | Probe: CTGTACTACGCATTGTATAC |  |  |
| *Strongyloides stercoralis* | dispered repetitive sequence | Fwd: TCCAGAAAAGTCTTCACTCTCCAG |  | 77 |
|  |  | Rev: TGCGTTAGAATTTAGATATTATTGTTGCT | [1] |  |
|  |  | Probe: TCAGCTCCAGTTGAACAACAGCCTCCAA |  |  |
| *Blastocystis* spp. | 18s rRNA | Fwd: TGGTCCGRTGAACACTTTGGAT |  | 120 |
|  |  | Rev: CCTACGGAAACCTTGTTACGACTTCA | [1] |  |
|  |  | Probe: CTTCCTCTAAATGRTAAGATT |  |  |
| *Ancylostoma duodenales* | ITS-2 | Fwd: GAATGACAGCAAACTCGTTGTTG |  | 72 |
|  |  | Rev: ATACTAGCCACTGCCGAAACGT | [1] |  |
|  |  | Probe: ATCGTTTACCGACTTTAG |  |  |
| *Enterobius vermicularis* | 5S rRNA | Fwd: CAAACAACTGCATCACCAATAAC |  | 100 |
|  |  | Rev: AGTGTAGAGCAATAAGCAGTAAAG | [2] |  |
|  |  | Probe: TACCAACAACACTTGCACGTCTCTTCA |  |  |
| *H. nana* | ITS1 | Fwd: CATTGTGTACCAAATTGATGATGAGTA |  |  |
|  |  | Rev: CAACTGACAGCATGTTTCGATATG | [1] | 88 |
|  |  | Probe: CGTGTGCGCCTCTGGCTTACCG |  |  |
| enteric 16s |  | Fwd: TGCAAGTCGAACGAAGCACTTTA |  |  |
|  |  | Rev: GCAGGTTACCCACGCGTTAC | [1] | 92 |
|  |  | Probe: CGCCACTCAGTCACAAA |  |  |
| PhHV | gB | Fwd: GGGCGAATCACAGATTGAATC |  |  |
|  |  | Rev: GCGGTTCCAAACGTACCAA | [1] | 89 |
|  |  | Probe: TATGTGTCCGCCACCATCT |  |  |
| *Yersinia enterocolitica* | *lytA* | Fwd: TGATTCACCAGCAGCAATAC |  |  |
|  |  | Rev: GGCATCATGAAAGGCGG | [1] | 156 |
|  |  | Probe: TGTCGGTTTCTCCTTCCAGG |  |  |
| *Heliobacter pylori* | *ureC* | Fwd: GACACCAGAAAAAGCGGCTA |  |  |
|  |  | Rev: AGCGCATGTCTTCGGTTAAA | [1] | 130 |
|  |  | Probe: TCACTAAAGCGTTTTCTACC |  |  |
| *Plesiomonas shigelloides* | *gyrB* | Fwd: CCGCCGTGAAGGCAAAG |  |  |
|  |  | Rev: GCTACCGGCTCACCCAGAT | [1] | 54 |
|  |  | Probe: CACACCCAAGAATAC |  |  |
| *Cyclospora cayetanensi* | 18s rRNA | Fwd: AAAAGCTCGTAGTTGGATTTCTG |  |  |
|  |  | Rev: AACACCAACGCACGCAGC | [1] | 118 |
|  |  | Probe: AAGGCCGGATGACCACGA |  |  |
| *Cystoisospora belli* | 18s rRNA | Fwd: ATATTCCCTGCAGCATGTCTGTTT |  |  |
|  |  | Rev: CCACACGCGTATTCCAGAGA | [1] | 90 |
|  |  | Probe: CAAGTTCTGCTCACGCGCTTCTGG |  |  |
| *Blastocystis* spp. | 18s rRNA | Fwd: TGGTCCGRTGAACACTTTGGAT |  | 120 |
|  |  | Rev: CCTACGGAAACCTTGTTACGACTTCA | [1] |  |
|  |  | Probe: CTTCCTCTAAATGRTAAGATT |  |  |
| *Enterocytozoon bieneusi* | SSU rRNA | Fwd: TGTGTAGGCGTGAGAGTGTATCTG |  | 103 |
|  |  | Rev: CATCCAACCATCACGTACCAATC | [1] |  |
|  |  | Probe: CACTGCACCCACATCCCTCACCCTT |  |  |
| *Encephalitozoon intestinalis* | ITS | Fwd: CACCAGGTTGATTCTGCCTGAC |  | 227 |
|  |  | Rev: CTAGTTAGGCCATTACCCTAACTACCA | [1] |  |
|  |  | Probe: CTATCACTGAGCCGTCC |  |  |
| *Balantidium coli* | ITS-1 | Fwd: TGCAATGTGAATTGCAGAACC |  |  |
|  |  | Rev: TGGTTACGCACACTGAAACAA | [1] | 92 |
|  |  | Probe: CTGGTTTAGCCAGTGCCAGTTGC |  |  |
| *Acanthamoeba* spp. | 18S rRNA | Fwd: CCCAGATCGTTTACCGTGAA |  | 171 |
|  |  | Rev: TAAATATTAATGCCCCCAACTATC | [3] |  |
|  |  | Probe: CTGCCACCGAATACATTAGCATGG |  |  |

**References**

1. Liu J, Gratz J, Amour C, Nshama R, Walongo T, Maro A, et al. Optimization of Quantitative PCR Methods for Enteropathogen Detection. PLoS One. 2016;11: e0158199. doi:10.1371/JOURNAL.PONE.0158199

2. Rudko SP, Ruecker NJ, Ashbolt NJ, Neumann NF, Hanington PC. Enterobius vermicularis as a Novel Surrogate for the Presence of Helminth Ova in Tertiary Wastewater Treatment Plants. 2017.

3. Qvarnstrom Y, Visvesvara GS, Sriram R, Da Silva AJ. Multiplex Real-Time PCR Assay for Simultaneous Detection of Acanthamoeba spp., Balamuthia mandrillaris, and Naegleria fowleri. J Clin Microbiol. 2006;44: 3589–3595. doi:10.1128/JCM.00875-06
