# Supplementary material for: Targeted pathogen profiling of ancient feces reveals common enteric infections in the Rio Zape Valley, 725–920 CE
Source: PLoS One. 2025 Oct 22;20(10):e0318140. doi: 10.1371/journal.pone.0318140 (PMC12543138; doi:10.1371/journal.pone.0318140)
Supplement: S2 Table — (DOCX) [file pone.0318140.s002.docx]

**Table S2.** TaqMan Array Card (TAC) performance and standard curve parameters**.**

| **Target** | **Target Gene** | **Slope** | **Y-intercept** | **R^2^** | **Efficiency** | **95% LOD† (copies/µL template)** |
| --- | --- | --- | --- | --- | --- | --- |
| enteric 16S | 16S | -3.309 | 38.881 | 0.998 | 101% | 1 |
| *Acanthamoeba* spp. | 18S rRNA | -3.3877 | 37.82 | 1.000 | 97% | 38 |
| *Ancylostoma duodenale* | ITS-2 | -3.3832 | 39.101 | 1.000 | 98% | 10 |
| *Ascaris lumbricoides* | ITS-1 | -3.4482 | 38.594 | 1.000 | 95% | 10 |
| *Balantidium coli* | ITS-1 | -3.3935 | 37.92 | 1.000 | 97% | 4 |
| *Blastocystis* spp. | 18S rRNA | -3.3196 | 40.64 | 0.997 | 100% | 4 |
| *Cystoisospora belli* | 18S rRNA | -3.3479 | 37.801 | 0.999 | 99% | 10 |
| *Cyclospora cayetanensi* | 18S rRNA | -3.3408 | 37.151 | 0.998 | 99% | 4 |
| *Campylobacter jejuni/coli* | *cadF* | -3.34178 | 38.27 | 0.999 | 99% | 35 |
| *Clostridium difficile* | *tcdB* | -3.4282 | 37.542 | 0.999 | 96% | 10 |
| *Cryptosporidium* spp. | 18S rRNA | -3.4033 | 37.983 | 0.999 | 97% | 1 |
| DNA control (phocine herpes virus) | *gB* | -3.315 | 37.009 | 0.998 | 100% | 10 |
| *Enterocytozoon bieneusi* | ITS | -3.2802 | 37.209 | 0.999 | 102% | 8 |
| *E. coli* O157:H7 | *rfbE* | -3.4568 | 37.976 | 1.000 | 95% | 4 |
| *Encephalitozoon intestinalis* | SSU rRNA | -3.3819 | 38.462 | 0.999 | 98% | 4 |
| *Enterobius vermicularis* | 5S | -3.4592 | 38.572 | 0.999 | 95% | 120 |
| EAEC (aaiC) | *aaiC* | -3.4241 | 38.15 | 0.999 | 96% | 10 |
| EAEC (aatA) | *aatA* | -3.4252 | 37.694 | 0.998 | 96% | 38 |
| *Entamoeba hystolytica* | 18S rRNA | -3.2775 | 37.994 | 0.996 | 102% | 10 |
| *Entamoeba* spp. | 18S rRNA | -3.2317 | 37.259 | 0.974 | 104% | 35 |
| EPEC (typical) | *bfpA* | -3.3772 | 37.465 | 0.999 | 98% | 10 |
| EPEC (atypical) | *eae* | -3.372 | 37.592 | 0.999 | 98% | 4 |
| ETEC (LT) | *LT* | -3.4638 | 47.637 | 0.990 | 94% | 485 |
| ETEC (STh) | *STh* | -3.3785 | 38.763 | 0.999 | 98% | 10 |
| ETEC (STp) | *STp* | -3.3548 | 37.266 | 0.999 | 99% | 4 |
| *Giardia* spp. | 18S rRNA | -3.4182 | 37.863 | 1.000 | 96% | 10 |
| *Hymenolepis nana* |  | -3.3804 | 38.248 | 1.000 | 98% | 4 |
| *Helicobacter pylori* | *ureC* | -3.4078 | 37.726 | 0.998 | 97% | 10 |
| *Shigella*/EIEC | *ipaH* | -3.3522 | 37.506 | 0.999 | 99% | 38 |
| *Necator americanus* | ITS-2 | -3.3686 | 39.806 | 1.000 | 98% | 7 |
| *Plesiomonas shigelloides* | *gyrB* | -3.4184 | 38.202 | 1.000 | 96% | 38 |
| *Salmonella* spp. | *invA* | -3.4191 | 38.427 | 1.000 | 96% | 4 |
| *Strongyloides stercolaris* | Dispersed repetitive sequence | -3.3316 | 37.527 | 0.999 | 100% | 4 |
| STEC (stx1) | *stx1* | -3.4083 | 39.883 | 1.000 | 97% | 120 |
| STEC (stx2) | *stx2* | -3.3697 | 38.328 | 0.967 | 98% | 160 |
| *Trichuris trichiura* | 18S rRNA | -3.3502 | 38.395 | 1.000 | 99% | 4 |
| *Yersinia enterocolitica* | *lytA* | -3.483 | 38.279 | 0.998 | 94% | 4 |

†95% LOD in gene copies per reaction calculated using methods from reference [1]

**References**

1. Stokdyk JP, Firnstahl AD, Spencer SK, Burch TR, Borchardt MA. Determining the 95% limit of detection for waterborne pathogen analyses from primary concentration to qPCR. Water Res. 2016;96: 105–113. doi:10.1016/j.watres.2016.03.026
