# Supplementary figures and images for: Targeted pathogen profiling of ancient feces reveals common enteric infections in the Rio Zape Valley, 725–920 CE
Source: PLoS One. 2025 Oct 22;20(10):e0318140. doi: 10.1371/journal.pone.0318140 (PMC12543138; doi:10.1371/journal.pone.0318140)

**Figure S1. Amplification and multicomponent plots**


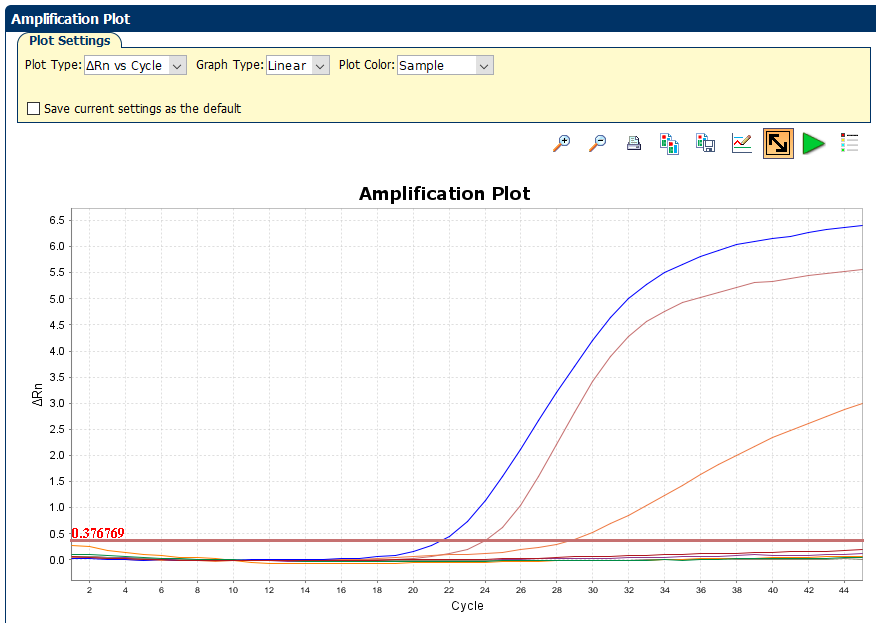


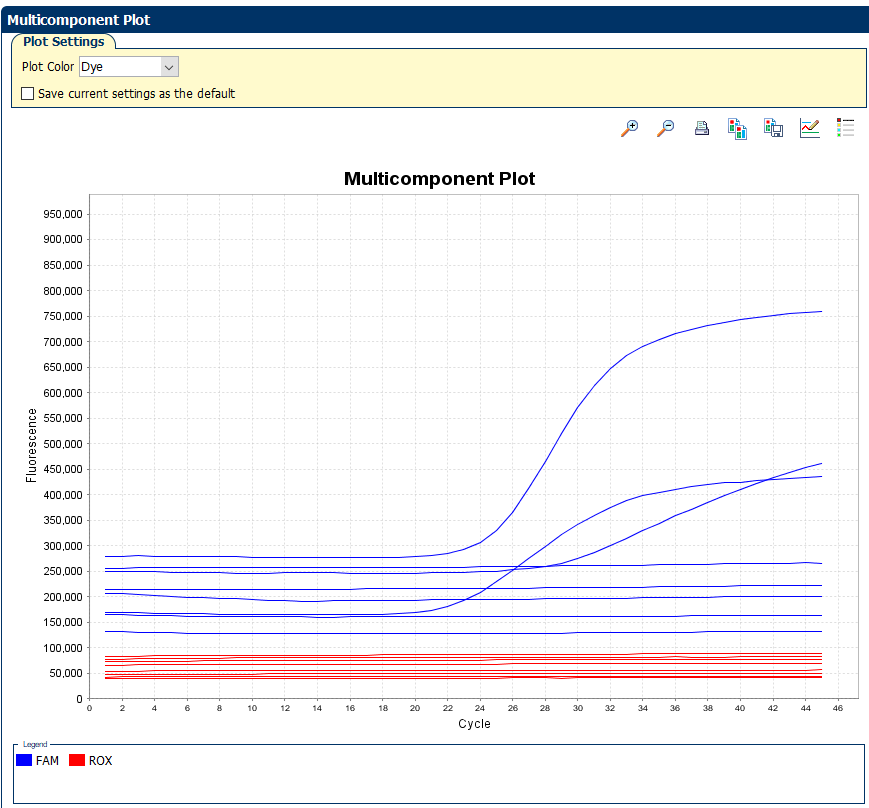

Supplement: S1 Fig — (DOCX) [file pone.0318140.s003.docx]
