# Supplementary material for: Targeted pathogen profiling of ancient feces reveals common enteric infections in the Rio Zape Valley, 725–920 CE
Source: PLoS One. 2025 Oct 22;20(10):e0318140. doi: 10.1371/journal.pone.0318140 (PMC12543138; doi:10.1371/journal.pone.0318140)
Supplement: S3 Table — (DOCX) [file pone.0318140.s004.docx]

Table S3. MIQE Checklist

| **ITEM TO CHECK** | **IMPORTANCE** | **CHECKLIST** |
| --- | --- | --- |
| **EXPERIMENTAL DESIGN** |  |  |
| Definition of experimental and control groups | **E** | Cross-sectional study with no intervention or control group |
| Number within each group | **E** | 10 paleofeces from a cave in the Rio Zape Valley |
| Assay carried out by core lab or investigator's lab? | D | Investigator's lab |
| **SAMPLE** |  |  |
| Description | **E** | 200 mg (see method’s section) |
| Volume/mass of sample processed | D | 200 mg |
| Microdissection or macrodissection | **E** | Not applicable |
| Processing procedure | **E** | See method’s section. |
| If frozen - how and how quickly? | **E** | Not frozen |
| If fixed - with what, how quickly? | **E** | Not fixed |
| Sample storage conditions and duration (especially for FFPE samples) | **E** | Shipped at ambient conditions. Stored at room temperature away a dark, cool, dry place |
| **NUCLEIC ACID EXTRACTION** |  |  |
| Procedure and/or instrumentation | **E** | See methods section |
| Name of kit and details of any modifications | **E** | Adapted from reference [1] |
| Source of additional reagents used | D | Qiagen PowerBead Tubes with garnet beads |
| Details of DNase or RNAse treatment | **E** | Not applicable |
| Contamination assessment (DNA or RNA) | **E** | One extraction negative control was included during each day of extractions |
| Nucleic acid quantification | **E** | Qubit 1X HS dsDNA Kit |
| Instrument and method | **E** | Qubit 4 Fluorometer |
| RNA integrity method/instrument | **E** | Not measured |
| Inhibition testing (Cq dilutions, spike or other) | **E** | Monitored amplification of spiked controls |
| **qPCR TARGET INFORMATION** |  |  |
| If multiplex, efficiency and LOD of each assay. | **E** | Table S2 |
| *In silico* specificity screen (BLAST, etc) | **E** | We BLASTed all assays to confirm specificity before ordering the custom TAC. |
| **qPCR OLIGONUCLEOTIDES** |  |  |
| Primer sequences | **E** | Table S1 |
| Probe sequences | D** | Table S1 |
| Location and identity of any modifications | **E** | No modifications |
| Manufacturer of oligonucleotides | D | ThermoFisher Scientific |
| **qPCR PROTOCOL** |  |  |
| Complete reaction conditions | **E** | 45°C for 20 min and 95°C for 10 min, followed by 45 cycles of 95°C for 15 s and 60°C for 1 min |
| Reaction volume and amount of cDNA/DNA | **E** | 6.66 µL of template, 31.33 µL molecular grade water, 2 µL inhibition control, with 60 µL of AgPath-ID™ One-Step RT-PCR Reagents |
| Primer, (probe), Mg++ and dNTP concentrations | **E** | All assays contained the same concentrations of primers (900 nanomolar) and probe (250 nanomolar). The Mg2+ and dNTP concentrations are not listed in the in the User Guide. |
| Polymerase identity and concentration | **E** | AmpliTaq Gold™ polymerase |
| Buffer/kit identity and manufacturer | **E** | AgPath-ID™ One-Step RT-PCR Reagents |
| Additives (SYBR Green I, DMSO, etc.) | **E** | No additives |
| Manufacturer of plates/tubes and catalog number | D | ThermoFisher Scientific |
| Complete thermocycling parameters | **E** | 45°C for 20 min and 95°C for 10 min, followed by 45 cycles of 95°C for 15 s and 60°C for 1 min |
| Reaction setup (manual/robotic) | D | Manual set-up in a disinfected dead air box (10% bleach with fifteen minutes of contact time, UV for fifteen minutes, and a final cleaning step with 70% ethanol) |
| Manufacturer of qPCR instrument | **E** | ThermoFisher Scientfic |
| **qPCR VALIDATION** |  |  |
| Evidence of optimisation (from gradients) | D | See references [2] and [3] |
| Specificity (gel, sequence, melt, or digest) | **E** | See references [2] and [3] |
| Standard curves with slope and y-intercept | **E** | Table S2 |
| PCR efficiency calculated from slope | **E** | Table S2 |
| r2 of standard curve | **E** | Table S2 |
| Evidence for limit of detection | **E** | Table S2 |
| **DATA ANALYSIS** |  |  |
| qPCR analysis program (source, version) | **E** | QuantStudio Real-Time PCR Software V1.2 CDC |
| Cq method determination | **E** | Manual thresholding |
| Results of NTCs | **E** | Reported in the results section |
| Justification of number and choice of reference genes | **E** | N/A |
| Description of normalisation method | **E** | Normalized to mass of paleofeces |
| Software (source, version) | E | R Studio V2.2.2 |

**References**

1. Hagan RW, Hofman CA, Hübner A, Reinhard K, Schnorr S, Lewis CM, et al. Comparison of extraction methods for recovering ancient microbial DNA from paleofeces. Am J Phys Anthropol. 2020;171: 275–284. doi:10.1002/ajpa.23978

2. Liu J, Gratz J, Amour C, Kibiki G, Becker S, Janaki L, et al. A laboratory-developed taqman array card for simultaneous detection of 19 enteropathogens. J Clin Microbiol. 2013;51: 472–480. doi:10.1128/JCM.02658-12

3. Liu J, Gratz J, Amour C, Nshama R, Walongo T, Maro A, et al. Optimization of Quantitative PCR Methods for Enteropathogen Detection. Chan KH, editor. PLoS One. 2016;11: e0158199. doi:10.1371/journal.pone.0158199
