# Supplementary material for: Targeted pathogen profiling of ancient feces reveals common enteric infections in the Rio Zape Valley, 725–920 CE
Source: PLoS One. 2025 Oct 22;20(10):e0318140. doi: 10.1371/journal.pone.0318140 (PMC12543138; doi:10.1371/journal.pone.0318140)
Supplement: S4 Table — (DOCX) [file pone.0318140.s005.docx]

Table S4. hCYTB484 human mtDNA primers and probe sequences.

| Oligonucleotide | Sequence (5’ to 3’) | Reference |
| --- | --- | --- |
| Fwd primer | CAATGAATCTGAGGAGGCTAC | Zhu, K.; Suttner, B.; Pickering, A.; Konstantinidis, K. T.; Brown, J. A Novel Droplet Digital PCR  Human MtDNA Assay for Fecal Source Tracking. Water Res. 2020, 183, 116085.  https://doi.org/10.1016/J.WATRES.2020.116085. |
| Rev primer | CGTGCAAGAATAGGAGGTG |  |
| Probe | ACCCTCACACGATTCTTTACCTTTCACT |  |
