# Supplementary material for: Targeted pathogen profiling of ancient feces reveals common enteric infections in the Rio Zape Valley, 725–920 CE
Source: PLoS One. 2025 Oct 22;20(10):e0318140. doi: 10.1371/journal.pone.0318140 (PMC12543138; doi:10.1371/journal.pone.0318140)
Supplement: Attachment — Submitted filename: Response to Reviewers.docx [file pone.0318140.s008.docx]

| Reviewer 1 Comment | Action Taken | Response |
| --- | --- | --- |
| L 27-28 : it is true that modern molecular tools such as PCR can be used in this kind of studies, but this is known since several decades now. So I’d tend to put the final emphasis of your abstract on something else you effectively demonstrated in your paper rather than this | Revised  Line 33-34. These results suggest that enteric infection may have been common among the Loma San Gabriel people, who lived in the Rio Zape Valley in Mexico during this period. | Agreed |
| L. 42 : Note that molecular identification of ancient pathogens or other past organisms has also, and still does, generated much debate. I’d suggest diminishing the idea of opposed methods and preferring the idea of proxy complementarity. Many studies showed how looking in parallel for molecular signatures, micro and macro remains, where actually much more informative rather than only aDNA or microscopy for instance. | We have worked this into the discussion. See lines 268-270 and 279-281. | Agreed. |
| L. 49-51 : once again, studying ancient pathogens from coprolites through their molecular signature is not something new in 2025 but your wording may suggest the contrary. See for example : Appelt S, Armougom F, Le Bailly M, Robert C, Drancourt M. Polyphasic analysis of a middle ages coprolite microbiota, Belgium. PLoS One. 2014 Feb 28;9(2):e88376. doi: 10.1371/journal.pone.0088376. PMID: 24586319; PMCID: PMC3938422. And it included microscopy-based diagnosis. | Revised  Line 56-58. Refinement of existing methods, combined with state-of-the-science molecular approaches, is increasingly being used for sensitive detection of fecal microbes in ancient feces [23,24]. | Agreed |
| L. 51 : could you be more specific on how such knowledge could expand our understanding of how peoples lived ? | Revised  Line 57-58. Such knowledge could expand our understanding of the burden of disease among ancients peoples and pathogen transmission among different groups. | We appreciate the feedback for additional clarity. |
| L. 61 : as you are not solely speaking to molecular biologists and microbiologists I think you should be more accurate on site description and chronological methods, even if it was previously published. You are also speaking to paleo/archaeo scientists in this paper. Note that you mentioned “previously collected […] material” (l. 57), a naive reader would then certainly understand that there is previous studies of these materials. You certainly should cite them here too. | We included 7 new paragraphs in the methods section starting on Line 63. | Agreed. |
| L.66-71 Could you please specify if this MSC is located in a clean room dedicated to aDNA analysis, or if it is at least dedicated to aDNA samples (meaning, it never saw any modern feces sample)? | We included this as a limitation.  Line 333. Fourth, we did not conduct molecular analyses in a clean room dedicated to ancient DNA analysis [47]. While we worked under sterilized BSL2 conditions with sterile single use reagents and observed no contamination in our negative controls, we cannot completely rule out the potential for cross contamination. | We worked under BSL2 conditions, using sterilized workspaces, and only used sterile single use plastics. |
| L.118-130 : Could you tell more in the core paper about these targets? How long are their amplicons, were they previously tested on modern samples? I think these informations should be made clear in the core text without having to dive in the supplementary files. | We included additional information in the methods  Line 179. The assays used were previously validated on modern fecal material with 100% sensitivity and 95%-100% specificity [28]. The average amplicon length for an assay was 116 bp (minimum = 54 bp, maximum = 238 bp) (Table S2). | We provide additional information in the text and supplemental. Further information on the validation of these assays can be found in reference 33 and 34. |
| L. 148-159: Wouldn’t it be appropriate to also test non-human targets? Is there any control performed with the surrounding sediment to distinguish the true feces’ content from surrounding leaching and environmental agents? | We establish in the methods that these 10 samples were previously identified as human origin based on dietary materials. See Table 1.  We added further discussion of the initial starting concentration of the human targets in modern fecal material.  Line 293. In our previous work with modern fecal material from school-age children, we found the concentration of enteric 16S rRNA was approximately 9,300 times greater than human mtDNA [50]. If these signals persisted at the same or similar rates, then this initial difference in concentration may explain why enteric 16S rRNA was observed above our limit of detection in each sample but human mtDNA was only detected in one sample. | We agree that this would be useful, but We don’t know the persistence of molecular signatures in desiccated soil.  In addition, these are surficial archaeological deposits. There were no surrounding sedimentary layers. The data regarding this 30-inch-deep midden was previously published in an extensive four-page table by Brooks and colleagues (1962). doi:10.2307/277801 |
| L. 201-203 : Did you have the opportunity to test the exact same coprolites (in order to know if some of them were positive under the microscope but not after PCR). | We have included Table 1 regarding dietary observations from these samples. | We did not have the opportunity to inspect the samples provided. Other samples from this cave had been inspected and 34% were positive for pinworm ova. |
| L. 213-216 : this also pleads for mentioning multi-proxy approaches I mentioned before. Far from diminishing your own approach, as you are potentially able to highlight it. | Revised  Line 279. This discrepancy further highlights the importance of using multiple complementary assays when interpreting evidence from ancient fecal material. | Agreed |
| L. 218-212 : Aside from the debate in itself, I would highly recommend to chose another wording for this sentence, as the detection of E. vermicularis per se does not support at all the absence of other helminths in pre-contact America. You are here substantially saying that most, if not all, published detections of Ascaris and hookworms from pre-contact America are misidentification. It’s a strong statement that should be based on case by case counter-identification based on published pictures, and not derived from the molecular detection of E. vermicularis within these 10 samples (I’m not working on this region/period, so don’t worry about me being triggered here). | Revised  Line 264. There is debate whether helminths other than pinworm were circulating among people in the Americas before the Columbian exchange began in 1492 [11]. The molecular detection of pinworm supports the use of qPCR assays, combined with microscopy and sequencing, to better understand which helminths were circulating in the Americas prior to the Columbian Exchange [23,24]. Given the potential for ova degradation, reported visual observations of hookworm and *Ascaris* ova in paleofeces from the Americas pre-1492 would be strengthened by further molecular validation. However, definitive conclusions should not be drawn from a small sample size of paleofeces from a single cave. | We appreciate the need for nuance here. |
| L. 227 : no other animals were tested if I’m correct (like, most commonly encountered cave dwelling mammals then and now). | We include additional information regarding the midden in the methods and revised this section of the discussion (see lines 282-303) |  |
| L. 229 : but you did not mention any test performed with sediment samples from the sedimentary layers surrounding these coprolites. | We added additional information to the methods section and cite the original reference (Ref 25, Brooks 1962) that describes excavation of the cave.. | See response to comment regarding Lines 148-159 |
| L. 233 : if most of these pathogens are shared between human and non-human hosts, then how could you conclude mostly about human behavior and waste management? I know there is a whole published archaeological background that could be used here to support your point, but you are not using it. So a naive reader can only think you decided a little bit arbitrarily it was human rather than non-human coprolites and pathogens, to document human waste management. | We have included additional information in the methods section, including Table 1. We also strengthened the human origin argument, but highlighting that *Enterobius vermicularis* is human specific.  Line 284. The presence of *Enterobius vermicularis* in most samples also provides strong evidence of human origin. *Enterobius vermicularis* is a human specific pathogen, though it can infect some species of apes[46–48]. | Thank you for this comment. The previous work with these samples provides evidence for human origin. |
| L. 235: Did Shigella and human mtDNA detection match the same coprolite? | Revised  Line 301. *Shigella* spp. is considered specific to humans, though the sample positive for *Shigella* spp. was negative for human mtDNA [44]. | It was from a different sample. |
| L. 252-261 : You did not provide any sequencing set that could be compared with environmental samples and assessed for aDNA damage patterns. Wouldn’t be considered as limitations? | Included in the limitations  Line 325. Damage and chemical modification patterns in ancient DNA can be observed via sequencing and bioinformatics analysis and could have been used to differentiate between endogenous ancient DNA and more recent potential contamination [47]. |  |
| As a whole I think this paper is interesting when suggesting that a well documented qPCR assay for modern samples and targeting a large variety of enteric pathogens could be used in paleodiagnosis. But as it is written at least, it is not at all integrated in archaeo/paleosciences. While it seems to highlight molecular and PCR based approaches as something new, it does not follow usual standards of authenticity in paleomicrobiology (typically providing sequence data showing damage patterns and control samples from the surrounding sedimentary layers, or at least discussions about depositional and post depositional process). It does not introduce at all the archaeological and sampling context, conditions and methods, and barely mentions previous studies from the same site. Finally, it tries to address the biological origin of the samples, but did not mention any real attempt to falsify the human origin hypothesis. It leaves me with the feeling that the paper, though technically interesting, did not, even remotely, attempt to address the archaeo/paleoscientific questions it was supposed to. | We have included additional information in the methods, with the appropriate citations of previous work on samples from this cave. | This is an important question. Our substantially revised methods and discussion now situate this manuscript within the previous archaeological work on this cave. We appreciate the reviewer’s comments that have helped us better situate this work within the field. |

| Reviewer 2 Comment | Action Taken | Response |
| --- | --- | --- |
| The authors address that they do not use metagenomic analysis, even though that is the norm in the ancient DNA field. Their arguments against it should be backed up by more data. For example in lines 246 and 247, the authors claim "Metagenomics  has a higher limit of detection than PCR, and sequencing may miss low abundance genomes" but do not provide a citation for this statement. I would be surprised if this were true. Especially as the authors do not discuss a large bais they will have when using PCR primers: ancient DNA is very short and most fragments will not be amplified using primers so they are able to see only the very well preserved, long DNA fragments using this method. I would rather say the strength of this method could be cost, which is important. NGS library preperation and sequencing to levels deep enough to detect these microbes would be far more expensive than this method. | We have included references to support this statement. We also revised to include the amplicon lengths in Table S1.  Line 314. Metagenomics has a higher limit of detection than PCR, and sequencing may miss low abundance genomes [21,22].  Line 181. The average amplicon length for an assay was 116 bp (minimum = 54 bp, maximum = 238 bp) (Table S2). | The amplicon lengths for our PCR assays are shorter than standard read lengths on the Illumina platform. |
| I would like a discussion of how the TaqMan kit they use could cause biases. This kit is designed to be used for cDNA and not genomic DNA and could thus cause biases due to non-specific amplification. | We acknowledge the potential for nonspecific amplification in the limitations.  Line 336. Finally, there is always a possibility of nonspecific amplification in PCR reactions, and if nonspecific amplification occurred, we may have overestimated the prevalence of the targets in our samples. However, we did not observe nonspecific amplification in our negative controls in this study or a previous study that used the same assays [41]. | We also provide citation 41, from a larger study of modern fecal material that used the same assays here. We did not observe non specific amplification in that study, which had a much larger number of negative controls. |
| . My biggest concern is the human mtDNA. Without sequencing it is impossible to claim that the human mtDNA comes from a true ancient source. In my experience with ancient DNA, samples themselves are often contaminated with modern human DNA. Thus a negative extraction control does not indicate that there is no potential modern human contamination. One would need to sequence this DNA and show patterns of ancient damage to be convincing that this comes from a human source. I think that making NGS libraries and sequencing are outside the scope of this study, however I would like this potential modern contamination discussed. | We have added this to the limitations  Damage and chemical modification patterns in ancient DNA can be observed via sequencing and bioinformatics analysis and could have been used to differentiate between endogenous ancient DNA and more recent potential contamination [47].  Line 213. Samples with less than three positive partitions were classified as negative to reduce the potential for false positives. | We acknowledge these limitations in the discussion section. We also now mention that our cutoff for positive partitions in our dPCR analysis is helpful to reduce false positives. |
| My last comment is minor: There is a typo in line 74 "The samples from were high in fiber [22]". I assume a word is missing? | Revised | Thanks for catching that. |

| Reviewer 3 Comment | Action Taken | Response |
| --- | --- | --- |
| Sequencing of Samples: The manuscript presents interesting findings, but an essential issue is the lack of sequencing for all amplified products. It is necessary to sequence all samples to confirm that the amplification is specific and accurately represents the target organisms. Without sequencing, there is a significant risk of false positives, particularly when working with microbial eukaryotes. | We acknowledge the potential for false positives in the limitations.  Line 336. Finally, there is always a possibility of nonspecific amplification in PCR reactions, and if nonspecific amplification occurred, we may have overestimated the prevalence of the targets in our samples. However, we did not observe nonspecific amplification in our negative controls in this study or a previous study that used the same assays [41]. | We acknowledge the limitations, but also provide citations that sequencings has a higher limit of detection than PCR (see line 314). As we expected low concentrations of our target, we selected PCR for its high sensitivity and specificity with low concentration samples. |
| Monitoring Evolution Over Time: The study could be greatly strengthened by incorporating a temporal analysis of the evolution of these organisms. This would add a valuable dimension to the research, providing insights into changes in pathogen profiles over time and improving the impact of the study. |  | We agree this is a very interesting potential analysis, but it was outside the scope of this study. |
| Language and Terminology: The manuscript would benefit from a thorough review of the language for clarity and readability. There are typographical errors that should be corrected. Additionally, statements such as "Blastocystis is a pathogen" should be carefully reconsidered. The pathogenicity of Blastocystis remains debated, and it would be more appropriate to refer to it as an organism commonly found in the gut rather than labeling it as a definitive pathogen. | Revised throughout | We revised throughout and refer to *Blastocystis* as a gut microbe in this versin. |
| Clarification of Pathogenicity Terminology: The manuscript states that Blastocystis is a pathogen. Given the ongoing debate regarding its pathogenicity, the authors should revise this statement to reflect current scientific consensus, such as referring to it as a "commonly detected gut microbe" rather than a definitive pathogen. | Revised throughout | See previous reponse |
| Grammar, Spelling, and Typographical Errors: The manuscript contains typographical and grammatical issues that should be corrected. A careful proofreading is necessary to improve readability and clarity. | Revised throughout | See previous comment on language and terminology. |
| Consistency in Terminology: Ensure that terminology such as paleofeces vs. coprolites is used consistently throughout the manuscript to avoid confusion. | Revised throughout | We now use the term paleofeces consistently. |
| PCR and qPCR Methodological Justification: The authors should provide a stronger justification for their choice of PCR/qPCR as the primary detection method without sequencing. If sequencing is not included, they should explicitly discuss the limitations and potential risks of false positives. | We include the risk of nonspecific amplification in the limitations and include references which demonstrate qPCR has a lower limit of detection compared to metagenomics.  Line 336. Finally, there is always a possibility of nonspecific amplification in PCR reactions, and if nonspecific amplification occurred, we may have overestimated the prevalence of the targets in our samples. However, we did not observe nonspecific amplification in our negative controls in this study or a previous study that used the same assays [41].  Line 314. Metagenomics has a higher limit of detection than PCR, and sequencing may miss low abundance genomes [21,22]. | See our response to your first comment. |
| Table 1: Ensure uniform formatting of columns and clearly indicate sample sizes for each prevalence calculation. Consider adding confidence intervals where applicable for prevalence values. Figures and Legends: | Revised for clarity. | Given a small sample size, 95% confidence intervals are not necessary here. |
| Figures should have more descriptive legends to ensure they are self-explanatory. If possible, improve resolution and clarity of any images, particularly those displaying results. Reference Updates: Ensure all references are up-to-date and correctly formatted according to PLOS ONE guidelines. | Revised for clarity. | See previous comment |

| Reviewer 4 Comment | Action Taken | Response |
| --- | --- | --- |
| Origin and Dating of Samples: o The authors do not specify how the dating of the samples was performed. Detailing this information would strengthen the study's credibility and accuracy in placing these samples in their correct historical context. | We included additional information.  Line 107. Three palaeofeces samples from the same proveniences represented by the Meade paleofeces series were submitted to DirectAMS for accelerator mass spectrometry radiocarbon dating measurements [30]. The three paleofeces samples were dated to 665-770, 770-953, and 778-986 CE. | Agreed |
| DNA Extraction: o The study does not mention the use of a dedicated clean room for ancient DNA (aDNA) extraction. Ancient DNA is highly degraded and prone to contamination from modern DNA. Best practices for aDNA extraction require a physically separated clean lab with HEPA-filtered positive-pressure air and full-body PPE (including face shields, lab coats, and gloves). The study should clarify whether these practices were implemented. | We have included this limitation in the discussion section.  Line 333. Fourth, we did not conduct molecular analyses in a clean room dedicated to ancient DNA analysis [49]. While we worked under sterilized BSL2 conditions with sterile single use reagents and observed no contamination in our negative controls, we cannot completely rule out the potential for cross contamination. | We worked under BSL2 conditions, using sterilized workspaces, and only used sterile single use plastics. |
| o Additionally, Uracil-DNA Glycosylase (UDG) treatment, which helps correct DNA damage from deamination, is not mentioned. If UDG was not used, the authors should acknowledge this limitation in their study. If it was used, the specific procedure should be described. | We included this limitation  Uracil-DNA Glycosylase (UDG) treatment has been developed to remove uracil residues from ancient DNA and repair the resulting abasic sites [48]. However, we did not apply these methods. | We mention this in the limitations. |
| o The authors should also provide more information about DNA purity (e.g., A260/A280 and A260/A230 ratios) and whether they assessed the quality and fragmentation of the DNA (e.g., using a Bioanalyzer or TapeStation). Without assessing DNA integrity, there is a risk that the DNA may be too degraded to amplify efficiently, potentially affecting the results. | We include these results now  Line 153. The average concentration of dsDNA in our extracts – measured via a Qubit dsDNA Assay Kit on a Qubit 4 Fluoremeter (ThermoFisher, Waltham, Massachusetts) – was 5.1 nanograms per microliter (range: 0.30 – 19.6). | We did not assess the fragmentation of the DNA. |
| . qPCR Methodology: o The study mentions using 38 forward and 38 reverse primers from IDT (Coralville, Iowa), but the selection and validation of these primers for use with degraded or ancient DNA are not described. The authors should explain if these primers were specifically tested for their ability to work with low-abundance or fragmented DNA. It would be helpful to include a discussion of how they ensured the primers' efficacy in amplifying ancient DNA sequences. | We provide further information on these assays in the methods and the supplemental information.  Line 179. The assays used were previously validated on modern fecal material with 100% sensitivity and 95%-100% specificity [28]. The average amplicon length for an assay was 116 bp (minimum = 54 bp, maximum = 238 bp) (Table S2). | We provided additional information, and further information on these assays can be found in the references provided (33, 34). |
| Digital PCR: o The use of digital PCR (dPCR) to detect human mitochondrial DNA (mtDNA) in paleofeces is a sound approach to confirm the human origin of the samples. However, modern human mtDNA contamination is a common issue in laboratories, as it is highly abundant. The authors should address the potential for contamination and explain how they mitigated the risk of false positives for human fecal origin. This could be crucial in ensuring the accuracy of their findings regarding the genuine ancient DNA sequences. | We acknowledge this in the limitations.  Line 333. Fourth, we did not conduct molecular analyses in a clean room dedicated to ancient DNA analysis [50]. While we worked under sterilized BSL2 conditions with sterile single use reagents and observed no contamination in our negative controls, we cannot completely rule out the potential for cross contamination. | We worked under BSL2 conditions, using sterilized workspaces, and only used sterile single use plastics. Further, we used a cutoff for positive partitions to reduce the potential for false positives (see line 214). |
