# Supplementary material for: Targeted pathogen profiling of ancient feces reveals common enteric infections in the Rio Zape Valley, 725–920 CE
Source: PLoS One. 2025 Oct 22;20(10):e0318140. doi: 10.1371/journal.pone.0318140 (PMC12543138; doi:10.1371/journal.pone.0318140)
Supplement: Attachment — Submitted filename: Response to Reviewers_R2.docx [file pone.0318140.s009.docx]

We thank the reviewers for their time and thoughtful comments. Line numbers reflect the tracked changes version of the manuscript.

| Reviewer Comment | Action Taken | Response |
| --- | --- | --- |
| 1. Sample Size and Statistical Justification While I understand the constraints of working with ancient material, the manuscript still lacks any form of quantitative justification for the sample size (n = 10), nor does it provide a clear explanation of the implications of such a limited dataset. There is no power analysis or alternative rationale. This omission weakens the strength of the claims, especially those that discuss prevalence or make broader epidemiological inferences. I strongly recommend softening these conclusions and clearly communicating the exploratory nature of the work. | We have softened the conclusion  Line 32  These results suggest that enteric infection may have been common among the Loma San Gabriel people, who lived in the Rio Zape Valley in Mexico during this period. | Our study objectives were to (1) apply modern stool based molecular diagnostics to ancient fecal samples; and (2) assess the enteric pathogen profile of these samples. We are not comparing between groups, or attempting and statistical analysis in this paper, so a sample size calculation is not necessary. |
| 2. Overinterpretation of Results Several conclusions—such as the suggestion that "enteric infections were common" among the Loma San Gabriel people—are not sufficiently supported given the data. This interpretation does not take into account:  the potential for false positives, especially with low-level amplification,  lack of authentication for ancient DNA (e.g., no sequencing or damage pattern analysis),  limited replication and variability in concordance between replicates,  and absence of confirmatory tests (e.g., amplicon sequencing or histological correlation).  These concerns are not fully addressed in the revised manuscript. | We have softened the conclusion  These results suggest that enteric infection may have been common among the Loma San Gabriel people, who lived in the Rio Zape Valley in Mexico during this period.  We mention the poor sensitivity of sequencing at low concentrations on Line 53.  Given the poor analytical sensitivity associated with metagenomic methods for targets present at low concentrations, analysis is limited to communities and generally not specific pathogens without deep sequencing [21,22]. | With a typical depth of sequencing, metagenomic sequencing is not reliable at distinguishing different species of the same genus, and less reliable at differentiating strains of the same species, as we have done here with pathogenic E. coli for example. qPCR is more sensitive than sequencing and the appropriate choice for identifying specific helminths and enteric pathogens in ancient feces. |
| 3. Limited Replication and Concordance The replication data show that 35% of targets were detected in only one of two or three replicates. This level of variability raises questions about reproducibility and the robustness of detections. While some variability can be expected in low-biomass, degraded samples, the extent observed here undermines confidence in the findings. This needs to be more transparently acknowledged in the main text and considered in the interpretation of prevalence data. | We added additional discussion to the limitation on Line 322  There are several limitations associated with this work. First, we analyzed a small number of samples from a single cave. An analysis of additional samples may have detected other pathogen-associated genes or a different prevalence of the genes we detected. We observed perfect concordance between replicates for most targets that we detected. Intra-stool heterogeneity has been demonstrated for enteric pathogen in modern feces.[56] We did not homogenize entire ancient fecal samples, which is common when working with fresh feces, and may have contributed to the heterogeneity in detection. | The level of heterogeneity in detection of specific pathogens in ancient feces is not well characterized. Heterogeneity is commonly observed in pathogen concentrations in fresh feces. |
| 4. Molecular Authentication of Ancient DNA The authors did not incorporate any ancient DNA authentication strategies—such as damage pattern analysis, uracil-DNA glycosylase treatment, or independent validation through sequencing—to demonstrate that the detected targets represent ancient rather than modern or environmental contamination. Given that the study’s novelty hinges on being the “first” to report certain pathogens in paleofeces, this omission is significant and weakens confidence in the findings. | We have revised to acknowledge this in the limitations on Line 331.  Damage and chemical modifications in ancient DNA—such as cytosine deamination—can be assessed through sequencing and bioinformatic analysis to help distinguish endogenous ancient DNA from modern contamination [57]. Uracil-DNA Glycosylase (UDG) treatment has been developed to remove uracil residues and repair resulting abasic sites[58]. While we did not employ these methods, they are primarily used in metagenomic studies and are less applicable to qPCR-based detection. However, the presence of deaminated nucleotides in ancient feces may have impaired primer and probe binding or amplification efficiency, potentially leading to underestimation of the prevalence of our target genes. | These enteric pathogen targets are not naturalized in the environment. In addition, sequencing of our PCR products would not have been able to differentiate between ancient DNA and modern DNA. |
| 5. Insufficient Biological Context for Detected Taxa The manuscript continues to list pathogen-associated genes without offering substantive biological interpretation. While additional references were added, the discussion remains superficial. For example, the ecological relevance, potential zoonotic transmission, or expected prevalence in ancient vs. modern contexts are not explored for many of the bacteria or protozoa. Without such context, the findings are descriptive rather than explanatory. | We mention that pinworm and *Shigella* are generally considered human specific. | We are hesitant to compare pathogen prevalence in ancient feces given that the decay rate of DNA in ancient fecal material is unknown and our detections likely underestimate the original prevalence in these samples. Work by Meade (see Table 1) and the detection of pinworm in most samples suggests these were human feces. We do not have evidence these were animals feces and did not analyze animal feces, so we are unable to speculate on zoonotic transmission in this context. |
| 6. Wording and Clarity Despite some edits, the manuscript continues to rely on vague or subjective language (e.g., “diverse,” “common,” “novel”). I recommend further revision to use precise, evidence-based language. The interpretation of results should be framed with appropriate caution given the study’s limitations. | We made additional revisions to the manuscript. | We appreciate the need for clarity. |
| 7. Controls and Contamination The authors note that 16S rRNA genes were detected in negative controls and attribute this to contamination of commercial reagents. While this is plausible and a known issue, it underscores the need for independent validation (e.g., sequencing amplicons or removing affected targets from interpretation). This issue is not sufficiently addressed in the discussion. |  | We appreciate the reviewer’s point regarding the need for caution when interpreting results potentially affected by contamination. We observed low-level amplification of the 16S rRNA gene in negative controls, which we attributed to well-documented background contamination in commercial reagents. it is unlikely that sequencing alone would allow us to differentiate between ancient DNA and trace modern contamination from reagents or the environment. The universal and conserved nature of 16S primers, combined with the lack of DNA damage signal in short amplicons, limits the interpretive power of sequencing in this specific context. |
| 8. Conclusions The authors conclude that modern molecular techniques are effective for detecting pathogens in paleofeces. While this may be true in principle, the methods applied here lack several key elements (e.g., authentication, replication consistency, host confirmation in most samples) that are critical in ancient DNA studies. The paper would benefit from reframing this conclusion as a proof-of-concept rather than an authoritative epidemiological report. | We have revised the manuscript and included additional discussion regarding the results. See lines 322-358. | While this study is limited in sample size, our results demonstrate the potential for applying sensitive, targeted molecular tools to detect a range of enteric pathogens in paleofeces using qPCR at the species level that would likely be missed by sequencing. We agree that future work that incorporates DNA damage profiling, clean room protocols, and broader comparative analyses on a larger sample size would be useful for robust epidemiological interpretation and authentication of ancient pathogen signatures. |

| Use of Class IIA Biological Safety Cabinet Instead of a Clean Room DNA extractions were performed in a Class IIA BSC, disinfected with 10% bleach, 70% ethanol, and UV light, rather than a dedicated aDNA clean room. Class IIA BSC Provides a sterile workspace with laminar airflow and UV sterilization but lacks positive pressure, HEPA filtration to remove airborne DNA, and physical separation of pre- and post-PCR areas. BSCs are designed for biosafety, not aDNA-specific contamination control. aDNA Clean Room Features positive pressure to prevent external DNA entry, HEPA-filtered air, UV-irradiated surfaces, and segregated pre- and post-PCR facilities to prevent amplicon carryover. These are essential for aDNA to avoid modern DNA contamination, which is abundant in microbial-rich environments like paleofeces. The BSC’s limitations likely contributed to the 16S rRNA contamination, as environmental DNA (e.g., from lab air, surfaces) could have entered during grinding or transfer. This is critical for aDNA, where low-yield, degraded samples are easily overwhelmed by modern DNA. | We now mention that the BSC was in a separate room from where PCR was performed.  We acknowledge this limitation on Line 348.  Further, we did not conduct molecular analyses in a clean room dedicated to ancient DNA analysis [60]. While we worked under sterilized BSL2 conditions with sterile single use reagents and observed no contamination in our negative controls, we cannot completely rule out the potential for cross contamination. | Unfortunately, we did not have access to a clean room, and we did not have the funds to construct a clean room for this study. We have acknowledged this limitation in the discussion section. |
| --- | --- | --- |
| - The study does not mention applying UV irradiation to paleofeces samples prior to DNA extraction, a critical step in aDNA protocols to degrade modern surface DNA contamination. UV irradiation (e.g., 254 nm, 30–60 minutes) is commonly used in aDNA studies to cross-link and degrade modern DNA on sample surfaces, reducing contamination risks from handling, excavation, or environmental exposure. Paleofeces, being organic and porous, are particularly prone to modern microbial DNA adhesion. | We added this limitation on Line 351.  We did not perform UV surface sterilization of the paleofeces, as enteric pathogens replicate exclusively in the gut and are not part of endogenous soil microbiota. Potential surrounding soil contamination during excavation was highly unlikely to introduce these organisms to paleofeces surfaces. | These enteric pathogen targets are not naturalized in the environment and would not have been introduced from the surrounding soil. |
| - The study relies on real-time PCR (TAC) and digital PCR fluorescence signals (Cq values, manual thresholding) without sequencing amplicons to confirm their identity. Sequencing (e.g., Sanger or next-generation sequencing) is essential in aDNA studies to verify that amplified products are ancient, not modern contaminants or non-specific amplifications. | We added additional detail on Line 316.  Metagenomics has a substantially higher limit of detection than PCR, and sequencing may miss low abundance genomes [21,22]. If genomes are similar, then bioinformatics pipelines may be unable to resolve species or genus-level details. | This was a qPCR study to detect specific enteric pathogen associated genes. It is unlikely that sequencing short amplicons that resulted from our PCR reactions would be able to differentiate between ancient DNA and modern contamination. |
| - The primers and extraction method were validated on modern feces (Ref 34), not aDNA, which is fragmented and chemically modified (e.g., deamination). This risks false negatives or reduced sensitivity. | We included this limitation on Line 339  While we did not employ these methods, they are primarily used in metagenomic studies and are less applicable to qPCR-based detection. However, the presence of deaminated nucleotides in ancient feces may have impaired primer and probe binding or amplification efficiency, potentially leading to underestimation of the prevalence of our target genes. | The extraction methods were validated on ancient feces. See Hagan et al. 2020 (reference 20). |
| - The absence of photographic documentation of the paleofeces (coprolites) in the study is a significant oversight, as visual evidence of their appearance could have provided critical clues about their probable origin (human vs. canine) based on shape, size, texture, or dietary inclusions, especially in the mixed human-canine Rio Zape midden (Ref 28) | See Table 1 for data on the dietary composition of these ancient feces | We agree this was a missed opportunity. Unfortunately, no photos of these samples exist. |
